# Implementation of simulation modelling to improve service planning in specialist orthopaedic and neurosurgical outpatient services

**DOI:** 10.1186/s13012-019-0923-1

**Published:** 2019-08-09

**Authors:** Nicole Moretto, Tracy A. Comans, Angela T. Chang, Shaun P. O’Leary, Sonya Osborne, Hannah E. Carter, David Smith, Tania Cavanagh, Dean Blond, Maree Raymer

**Affiliations:** 1 Centre for Health Services Research, Faculty of Medicine, The University of Queensland, Princess Alexandra Hospital campus, Woolloongabba, Queensland 4102 Australia; 2 Metro North Hospital and Health Service, Royal Brisbane and Women’s Hospital, Butterfield Street, Herston, Queensland 4029 Australia; 3 School of Health and Rehabilitation Sciences, Faculty of Health and Behavioural Sciences, The University of Queensland, St Lucia, Queensland 4067 Australia; 4 School of Nursing and Midwifery, Faculty of Health, Engineering and Sciences, University of Southern Queensland, Ipswich, Queensland 4305 Australia; 5 Australian Centre for Health Services Innovation, School of Public Health and Social Work, Institute of Health and Biomedical Innovation, Queensland University of Technology, Kelvin Grove, Queensland 4059 Australia; 6 West Moreton Health, Ipswich, Queensland 4305 Australia; 7 Cairns and Hinterland Hospital and Health Service, Cairns, Queensland 4870 Australia; 8 Gold Coast Health, Southport, Queensland 4215 Australia

**Keywords:** Implementation, Discrete event simulation, Orthopaedics, Neurosurgery, Physiotherapy, Hospital, Costs, Economic evaluation

## Abstract

**Background:**

Advanced physiotherapist-led services have been embedded in specialist orthopaedic and neurosurgical outpatient departments across Queensland, Australia, to ameliorate capacity constraints. Simulation modelling has been used to inform the optimal scale and professional mix of services required to match patient demand. The context and the value of simulation modelling in service planning remain unclear. We aimed to examine the adoption, context and costs of using simulation modelling recommendations to inform service planning.

**Methods:**

Using an implementation science approach, we undertook a prospective, qualitative evaluation to assess the use of discrete event simulation modelling recommendations for service re-design and to explore stakeholder perspectives about the role of simulation modelling in service planning. Five orthopaedic and neurosurgical services in Queensland, Australia, were selected to maximise variation in implementation effectiveness. We used the consolidated framework for implementation research (CFIR) to guide the facilitation and analysis of the stakeholder focus group discussions. We conducted a prospective costing analysis in each service to estimate the costs associated with using simulation modelling to inform service planning.

**Results:**

Four of the five services demonstrated adoption by inclusion of modelling recommendations into proposals for service re-design. Four CFIR constructs distinguished and two CFIR constructs did not distinguish between high versus mixed implementation effectiveness. We identified additional constructs that did not map onto CFIR. The mean cost of implementation was AU$34,553 per site (standard deviation = AU$737).

**Conclusions:**

To our knowledge, this is the first time the context of implementing simulation modelling recommendations in a health care setting, using a validated framework, has been examined. Our findings may provide valuable insights to increase the uptake of healthcare modelling recommendations in service planning.

**Electronic supplementary material:**

The online version of this article (10.1186/s13012-019-0923-1) contains supplementary material, which is available to authorized users.

Contributions to the literature

- Simulation modelling is a valid decision-support tool that enables decision-makers to plan efficient services to meet the rising demand for healthcare services.
- Due to the limited research on the use and implementation of modelling results in healthcare, little is known about the factors that influence the adoption of modelling results to inform service planning.
- Our findings provide useful insights into how and why decision makers adopt modelling results and its value in healthcare. These findings can be used to inform implementation strategies to increase the use of modelling in service planning.

## Introduction

Musculoskeletal conditions place an enormous burden on health services in Australia and worldwide [1]. With their prevalence in Australia set to rise [2], specialist orthopaedic and neurosurgical outpatient services face the challenge of reducing the demand-capacity gap to ensure patients are seen within clinically recommended timeframes. As the majority of patients referred to these services do not require surgical intervention [3], physiotherapist-led models of care have been introduced as a way of increasing the availability of non-surgical care options for patients. Advanced physiotherapist-led models have been embedded in the majority of public orthopaedic and neurosurgical outpatient services across Queensland, Australia, as they have been shown to provide high-quality patient care and be cost-effective compared to medical specialist-led models of care in managing orthopaedic demand [4, 5]. However, some of these services do not have the optimal combination of medical specialist and physiotherapist-led services to address current and projected future demand.

Many countries faced with budget restrictions routinely use economic evaluation to ensure the efficient and effective use of healthcare resources [6, 7]. Economic evaluations often incorporate healthcare modelling techniques to assess cost-effectiveness (value for money) of healthcare interventions to inform reimbursement decisions [7, 8]. Modelling results are used to evaluate the affordability of healthcare interventions and their economic impact on healthcare budgets [9]. Simulation modelling, such as discrete event simulation, has been used to support medical (e.g. cost-effectiveness analysis of healthcare interventions) and health policy decisions (e.g. prevention and screening programs, spread of infectious diseases) and has been applied extensively in the area of healthcare operations and system design [10, 11]. The ability of discrete event simulation to simulate patient journeys through the care system [12] and to incorporate capacity and resource constraints makes it an effective tool to manage and forecast resources (e.g. manage and predict bed capacity) and improve service flow (e.g. reduce queues or waiting times) in complex healthcare systems [6, 11, 13, 14]. Discrete event simulation models are built to support operational decision-making, resource allocation and optimisation and planning decisions [15] that has application in service planning for challenging health problems such as musculoskeletal conditions [5, 16]. Discrete event simulation has been used successfully to identify the most efficient and cost-effective scale and professional mix of services required to achieve waiting time targets in orthopaedic and neurosurgical outpatient services [5, 16].

Although simulation modelling has been shown to be a valid, decision support tool for informing service planning [10, 13], little is known about the use and implementation of its results in healthcare [17–20]. Most simulation modelling publications simply report modelling results [21] with only a few reporting its implementation strategy [22–25]. Furthermore, the value of simulation modelling in a healthcare context remains unclear due to limited economic evaluations of modelling implementation [6, 19, 23, 24, 26]. More research is needed to explore and better understand the factors that influence the use, implementation and the value of implementing simulation modelling in service planning.

Engaging key stakeholders in the simulation modelling process is considered critical to the success of the implementation [27]. This study sought to evaluate the effectiveness of engaging stakeholders early and involving them in the simulation modelling process with support from implementation leaders. In collaboration with stakeholders, we sought to facilitate the exploration of feasible ‘what if’ scenarios to identify potential outcomes of different healthcare strategies that could be used as a basis for making changes to service delivery [14].

To address the deficits in the healthcare literature concerning simulation modelling implementation, we undertook an implementation study that had two purposes. In part I, using an implementation science approach, we aimed to (i) assess the use of the simulation modelling recommendations in business cases and (ii) explore stakeholder perspectives about the role of simulation modelling in service planning, including identifying the contextual factors that influenced the use of simulation modelling recommendations, for service re-design of musculoskeletal outpatient services. In part II, we aimed to examine the costs of developing and implementing a simulation modelling approach to inform service planning.

## Method

Part I of this study involved a prospective qualitative evaluation. This evaluation assessed the use of the simulation modelling recommendations and explored stakeholder perspectives regarding the use of simulation modelling for musculoskeletal service re-design in specialist orthopaedic and neurosurgical outpatients in three Queensland health districts. Part II of the study involved a prospective, cost analysis to investigate the costs of developing a simulation model and using the model’s results for musculoskeletal service re-design.

The Gold Coast Hospital and Health Service Human Research Ethics Committee provided multi-site approval for this study (reference number HREC/16/QGC/205). The Queensland University of Technology provided administrative ethics approval (reference number 1600000794).

### Setting

Five outpatient services (three orthopaedic and two neurosurgical) participated in the study. The services are located across three public health districts in Queensland, Australia, and serve 23% of Queensland’s population (approximately 1.1 million people). In the Queensland public health system, non-emergency patients that require specialist outpatient care are referred by their general practitioner to the specialist outpatient department of their nearest public hospital. All referrals received by the specialist outpatient department are assessed, triaged and categorised based on their level of clinical urgency. Patient referrals are categorised as urgent (category 1), semi-urgent (category 2) and non-urgent (category 3) with recommended timeframes for an initial outpatient consultation within 30, 90, and 365 days, respectively. Once categorised, all patients are added to the relevant specialist outpatient waitlist to wait for an initial outpatient consultation.

In late 2015, the five participating services had a combined outpatient orthopaedic and neurosurgical waiting list of approximately 9100 and 2400 people, respectively. Patient wait times for initial outpatient consultations varied across the five services with approximately 38% of patients waiting longer than clinically recommended by late 2016 (Additional file 1) [28, 29]. Median wait times across the five services at baseline had reached 30 days (range 2–454), 327 days (range 22–674) and 462 days (range 1–2311), for urgency categories 1, 2, and 3, respectively [30–32]. These participating services were chosen based on an identified gap between referral demand and capacity in their outpatient services and a likely sub-optimal professional mix of services to manage demand.

Planning for new services, or modifications to existing specialist orthopaedic and neurosurgical outpatient services, involves preparation of a business case by the relevant stakeholders which is submitted for consideration by the health district’s executive. The business cases outline evidence of the problem, benefits of the proposal, solutions, recommendations and costs.

### Simulation modelling intervention

We built five clones of a previously constructed discrete event simulation model that simulate orthopaedic and neurosurgical outpatient services. We adapted each model to reflect the local variations of each service. Comprehensive details of the modelling software and pathways used and refined in two previous projects have been published [5, 16]. We populated each model with extensive local service-specific data. We designed the models to determine the optimal scale and combination of medical specialist and physiotherapist-led services required to efficiently manage demand over 5 years, with the target of almost all patients being seen within clinically recommended timeframes for their urgency category. We developed the models as decision support tools to help inform service planning. We performed scenario analysis to allow decision makers to test the likely impact of making a variety of different service changes, before deciding whether to implement any changes to the scale and professional mix of services.

The simulation model results indicated that if growth in demand continues as forecast and service capacity remains unchanged over 5 years, waiting lists for orthopaedic and neurosurgical outpatient services would grow across the three sites. This would result in the majority of semi-urgent and non-urgent patients breaching target wait times. The modelling identified that under the current conditions, expanding the overall scale and maximising the use of physiotherapist-led services would be recommended for all five services to efficiently achieve a target of 80–99.9% of patients being seen within the clinically recommended wait time targets at the end of the 5 years.

### Implementation strategy

The research team developed and led the multi-stage simulation modelling implementation strategy across the five services at the three health districts.

#### Stage 1: Stakeholder engagement, model development and initial modelling results

An implementation leader was appointed at each health district. The leaders were associate investigators on the project and were the directors of physiotherapy at each site. The implementation leader at each health service identified key stakeholders, with whom we worked closely to obtain local data and to confirm the model structure, parameters and relevant outputs. We presented the initial modelling results (i.e. base case and optimisation), including the model parameters and assumptions, to the key stakeholders at each site. Stakeholders confirmed the modelling results were representative of their outpatient services. We modified the model as required.

#### Stage 2: Exploration of feasible scenarios

We worked with key stakeholders to explore a range of feasible scenarios, which involved testing a variety of possible changes to both the scale and professional mix of services. We used the model to predict the likely impact of making the different changes to service configurations within their health district.

#### Stage 3: Changes to service delivery

Stakeholders were able to use the modelling results as a basis for either developing a business case for service changes to be submitted to the health district’s executive or for implementing strategies to influence the mismatch between demand and service capacity in ways which mitigated the need for additional investment.

### Part I—Qualitative evaluation methods

#### Qualitative stakeholder focus groups and participants

We undertook an evaluation of the simulation modelling implementation at the three health districts. Our implementation evaluation was conducted and reported in accordance with the Standards for Reporting Implementation Studies (StaRI) checklist (Additional file 2). We conducted two rounds of focus groups using a semi-structured question guide, with probing questions relevant to each health district. We conducted the initial round of focus groups prior to developing the simulation model (September to October 2016). We conducted the second round of focus groups approximately 10 months later after presenting the final modelling results (July to August 2017) to align with the outcome announcements of business case decisions for the 2017/18 financial year. The implementation leaders identified and invited key stakeholders from each health district to participate in the focus groups. Relevant stakeholders included lead clinicians from participating services (i.e. medical specialists, surgical specialists, physiotherapists), staff responsible for relevant services and departments (e.g. service directors, department directors) and members of the executive management team responsible for broad service areas and portfolios within the health districts (e.g. clinical directors, executive directors).

All stakeholders provided written informed consent to participate. An independent facilitator conducted the focus groups. A research assistant was a note taker. No research team members were present. The face-to-face focus groups were held onsite at each of the three health districts and ranged from 40 to 50 min. The focus groups were audio recorded, transcribed verbatim and de-identified.

#### Qualitative data collection and conceptual framework

We used the consolidated framework for implementation research (CFIR) [33] to inform the research design and to guide question development, qualitative coding and analysis. The CFIR is a practical structure for understanding complex, interrelating, multi-level and transient states of elements that could influence implementation in the real world [33]. The framework was developed from a synthesis of published implementation theories and includes 39 constructs across five domains: intervention characteristics, outer setting, inner setting, characteristics of individuals and process [33].

The research team selected a subset of CFIR constructs considered likely to influence the use of simulation modelling recommendations to inform decision making. The team selected the constructs a priori based on a review of the published literature and on their knowledge of public outpatient settings. The constructs used to inform the focus group questions were readiness for implementation (inner setting), implementation climate (inner setting), knowledge and beliefs about the intervention (characteristics of individuals), external policy and incentives (outer setting) and evidence strength and quality (intervention characteristics).

#### Qualitative data analysis

A qualitative researcher (facilitator, JG) and an implementation scientist (SO) analysed the transcriptions manually and using NVivo 10 software [34], respectively. The researchers used the constant comparative method as described by Sopcak and colleagues [35], drawing upon the early work of Glaser and Strauss [36]. The analysts independently coded the transcripts line by line, first inductively (open coding) and then deductively (using CFIR constructs); grouped the codes into categories (axial coding); compared and refined codes; and discussed the emerging higher-level themes (selective coding). Coding inconsistencies were addressed through team discussion.

#### Rating the CFIR constructs

Two researchers (NM, AC) independently assigned ratings reflecting the valence for each construct for each site based on the qualitative results. The ratings indicated a positive (+), negative (−) and mixed (X) influence of each construct on the use of the simulation modelling recommendations. Rating inconsistencies were addressed through discussion.

#### Quantitative measure of implementation success

Defining the measure of implementation success enables the effectiveness of implementation strategies to be compared across studies [37]. This study focussed on the use of simulation modelling recommendations to inform service planning in specialist outpatient services. We measured the implementation effectiveness using *adoption*, defined as the intention, initial decision or action to try or employ an innovation [37]. Adoption was demonstrated by the inclusion of simulation modelling recommendations for service changes into a business case for consideration by the health district’s executive.

#### Analysis of CFIR constructs

We created a matrix that listed each of the sites (columns) and the corresponding ratings for each of the constructs (rows). As described by Damschroder and Lowry [38], we compared the ratings of the CFIR constructs and identified patterns in the ratings that distinguished the two high implementation effectiveness sites with the one mixed implementation effectiveness site. We categorised the constructs as strongly, weakly or not distinguishing constructs between high and mixed implementation sites.

### Part II—Cost of implementation

We estimated the costs associated with developing and adapting the simulation model at each site. Project staff and relevant stakeholders completed a self-report activity log (Additional file 3) estimating the number of hours spent on activities at the end of each of the three stages of the simulation modelling implementation strategy (described above). Staff time was valued using 2016–2017 financial year salary data including employer on-costs. Contractor time and costs were taken directly from the invoices received for activities related to the project. We excluded costs associated with the qualitative evaluation, overheads, computers and modelling software licences.

A separate economic evaluation was conducted as a case study using data from one of the participating outpatient services. The economic evaluation accounted for the costs and outcomes under a scenario where the recommendations from the simulation model were applied in practice. This analysis was based on a business case that adopted the simulation modelling recommendations, and was approved by the health district’s executive for the 2017/2018 financial year. Further details of this case study are provided in Additional file 4.

## Results

### Part I—Qualitative evaluation

Twenty-nine stakeholders participated in the initial focus groups (site A, 10; site B, 8; site C, 11). One participant withdrew from the study following the initial focus group. Twenty-four stakeholders participated in the final focus groups (site A, 10; site B, 7; site C, 7). One participant at site C was unable to attend the final focus group and was interviewed separately.

#### Implementation effectiveness

Four of the five services (80%) demonstrated adoption as evidenced by the inclusion of recommendations based on modelling findings into business cases for service changes (Table 1). For all five services, modelling identified that an increase in the scale of services delivered and the proportion of physiotherapist-led activity would be required to efficiently meet waiting time targets. We categorised site A and site B as high implementation success sites, as their participating services incorporated modelling results into business cases for service changes. We categorised site C as a mixed implementation success site. This was because one of its participating services incorporated modelling results into a business case for service changes while its other participating service did not incorporate modelling results into a business case as the submission was deferred.

**Table 1:** Details of implementation effectiveness for the five services across the three sites

Site	Site A		Site B	Site C	
	High implementation success site		High implementation success site	Mixed implementation success site	
Service	A-1	A-2	B-1	C-1	C-2
Implementation effectiveness	High	High	High	High	Low
Adoption (i.e. business case submitted)	Yes	Yes	Yes	Yes	No
Business case outcome	Approved^a^	Approved^a^	Implemented corrective strategies; maintained watching brief	Approved^b^	Not applicable

^a^Permanent funding

^b^Temporary (financial year 2017–18) and recurrent (financial year 2018–19) funding

At the time of writing this paper, the outcomes of the business cases were known and health districts had enacted several service changes based on modelling recommendations. At site A, the executive approved both business cases for permanent funding. At site B, the service managers immediately implemented efficiency strategies, allowing them to maintain a watching brief on the business case to determine if additional changes to service delivery are required. At site C, the executive approved the business case for temporary additional funding at one service, which was made recurrent in the following financial year. The second service at site C focussed on optimising service efficiency before considering investment in additional resources.

#### Evaluation using CFIR

Ratings of the CFIR constructs are provided in Table 2. Of the six CFIR constructs examined, four constructs distinguished between the high and mixed implementation effectiveness sites. Findings from the qualitative analysis are presented below.

**Table 2:** Ratings assigned to CFIR constructs by site

Site	High implementation		Mixed implementation	
	A	B	C	
Intervention characteristics domain				
Evidence strength and quality	+	+	+	
Outer setting domain				
External policy and incentives	+	+	–	**
Inner setting domain				
Implementation climate				
Tension for change	+	+	–	**
Readiness for implementation				
Leadership engagement	+	+	–	**
Available resources	–	–	–	
Characteristics of individuals domain				
Knowledge and beliefs about the intervention	+	+	M	*

The valence of each construct is represented by ratings showing a positive (+), negative (−) and mixed (M) influence on the use of simulation modelling recommendations. Constructs were characterised as strongly (**) and weakly (*) distinguishing between high and mixed implementation sites

##### Distinguishing constructs

Two of the four constructs that distinguished between high and mixed implementation effectiveness fell within the inner setting domain: implementation climate (tension for change) and readiness for implementation (leadership engagement). The other two distinguishing constructs related to the outer setting domain (external policy and incentives) and characteristics of individual domains (knowledge and beliefs about the intervention).

Tension for change (perceived need for current situation to change) at all sites was primarily driven by the current status of the waiting lists and concerns about demand and population growth. Stakeholders at the mixed implementation site reported being weary of change and wary of any changes proposed by those outside of the organisation. This is in contrast to stakeholders at the high implementation sites who reported a strong tension for change, stating that they were likely to implement service changes based on modelling results as change was considered inevitable.

We’ve got to change or make some changes, whatever that looks like [site B, high implementation success site].

At the two high implementation sites, leadership engagement was demonstrated by the inclusion of all relevant stakeholders in the modelling process from the beginning, as they believed that early and continued staff involvement would increase the likelihood of the modelling being accepted. At the mixed implementation site, the executive and medical staff had limited engagement in the modelling project, which may have negatively influenced the acceptance of the modelling recommendations despite the compelling modelling findings. This is illustrated in the exemplar comment:

[The executive] didn’t actually try and understand [the modelling project] [site C, mixed implementation success site].

The influence of the outer setting was also identified as a significant success factor. The health services sit within a larger public health organisation, and consequently, external policy and incentives, such as budget cycles and external priorities, influenced stakeholders’ beliefs that simulation modelling would be of value if the modelling results were timely. Modelling was considered timely at the high implementation sites ‘as we do our master planning’ [site B, high implementation success site]. The timing of the modelling at the mixed implementation site may have affected its effectiveness, as evidenced by the comment that modelling was a ‘great thing at the wrong time’ [site C, mixed implementation success site]. The external policy environment (lack of forward planning) and priorities (managing and addressing demand, waiting lists and growth forecasts) were similar across the sites. Budget and waiting lists were considered the primary factors driving all decisions related to service changes.

Knowledge and beliefs in the intervention was a distinguishing factor at the two high implementation sites, where stakeholders had improved knowledge about modelling and its applications at the end of the project and placed a higher value on modelling compared to non-engaged stakeholders at the mixed implementation site. Stakeholders at high implementation sites believed modelling encouraged more robust, strategic and longer-term planning. Stakeholders reported that modelling facilitated better communication, focussed their attention on key issues and provided confidence in their service planning. Modelling was considered to be of most value when it provided evidence for solutions to issues considered a high priority for the health district, rather than just putting resources ‘where the [squeaky] wheel … is’ [site B, high implementation success site].

##### Non-distinguishing constructs

The two constructs that did not distinguish implementation effectiveness were evidence strength and quality and available resources. Stakeholders across all sites reported a high level of trust and confidence in the modelling inputs, assumptions and results which they stated was due to the model being populated with local, context-specific data that was validated by the stakeholders, ‘Validation is the key’ [site A, mixed implementation success site]. Resource availability was a concern for all sites, as stakeholders reported that funding to change services, based on modelling recommendations, was unlikely to be made available. Stakeholders questioned the value of commencing the modelling project if no funding was available for implementation.

The higher-level decisions are always driven by dollars so there might be a willingness but not a capacity [site A, high implementation success site].

#### Non-CFIR (emergent) constructs

Three additional constructs not able to be mapped to CFIR emerged from the qualitative analysis.

##### Evidence for advanced physiotherapist-led services

Stakeholders across all sites were positive about the value of advanced physiotherapist*-*led care in specialist outpatient services, believing that patients have received ‘good outcomes’ from their physiotherapist-led services [site A, high implementation success site].

##### Autonomy/locus of control

There was a sense across all sites that stakeholders were not fully able to influence decisions and had minimal control over the decision to make service changes. Several stakeholders referred to decisions being made ‘somewhere in that space’ between clinicians and the executive [site C, mixed implementation success site].

##### Economic benefit of undertaking modelling

Stakeholders at the high implementation sites were interested in whether the modelling was a cost-effective tool for service planning, stating ‘it’s worth [the investment] to get the data right to use this model’ [site A, high implementation success site].

### Part II—Cost of implementation

A detailed breakdown of staff time and costs associated with each stage of the simulation modelling implementation strategy is provided for each of the five participating services in Additional file 5. Activity logs indicated that an average of 336 h of staff time was spent on the simulation modelling study at each service. Allied health professionals, including mostly project team members, implementation leaders and stakeholders representing the physiotherapist-led services, accounted for 55% of this time.

Mean costs associated with staff time and travel for each stage of the simulation modelling implementation strategy across the five services are summarised in Table 3. The average cost per site was AU$34,553 (standard deviation = AU$737) across the three stages, with approximately 77% of total costs incurred during stage 1. The variance in costs between services was relatively minor.

**Table 3:** Costs of simulation modelling implementation strategy across the five participating orthopaedic and neurosurgical services

Stage	Costs (AU$)			
	Mean	SD	Minimum	Maximum
Stage 1	26,561	1002	25,325	27,644
Stage 2	4895	400	4334	5247
Stage 3	3097	1414	983	5088
Total	34,553	737	33,744	35,700

*SD* standard deviation. All costs are in Australian dollars. Costs were valued using 2016/2017 financial year salary data

## Discussion

This study has shown that the development of a simulation model and implementation of its results were highly effective (80% uptake) in changing the scale and mix of services to be delivered. This success rate is significantly higher than estimates in the literature, reported at between 5.3 and 30% in health care [17–21, 39] and up to 57% in outpatient services [39].

Our findings highlight the importance of the perceived need for change amongst stakeholders and a leadership team willing to engage all key stakeholders (leadership engagement) throughout the implementation process. Leadership engagement was found to enhance stakeholders’ knowledge and beliefs about the intervention. The impact of the external organisational environment of the outer setting was identified, particularly in relation to highlighting the importance of timing the modelling to support key decisions.

These findings are consistent with previous studies showing that tension for change and leadership engagement are important factors for the successful implementation of simulation modelling results [17, 22, 25, 27, 40–42]. Previous research highlights the importance of making sure the system being modelled is in need of a change or a decision [17, 26]. The strong tension for change at the high implementation sites is reflected in the high proportion of patients breaching wait time targets at site A (i.e. at baseline, 74% and 78% patients were waiting longer than clinically recommended in the orthopaedic and neurosurgical service, respectively) (Additional file 1). At site B, tension for change was reflected in its projected population growth, which is the largest of any health district in Queensland [43].

Researchers have emphasised the need to ensure decision makers are involved throughput the project, cautioning that modelling is likely to fail without interest and engagement from key decision makers [44]. Previous studies have demonstrated the link between leadership engagement and stakeholder knowledge. Involving stakeholders in the modelling process has been shown to enhance stakeholders’ belief in the value of modelling in supporting decision making and promote a greater understanding of the model and the problem itself [13, 22, 24, 45–47]. The in-depth understanding of the problem gained during the modelling process has been shown to increase the likelihood of successful implementation [20, 21, 48]. Simulation modelling has been shown to support healthcare decision making through promoting communication and fostering collaboration amongst stakeholders [40, 46, 49–52]. Previous studies suggest that the timing and responsiveness of the modelling results to support decisions are important considerations in overcoming implementation issues [17, 26].

Our findings show that evidence strength and quality and available resources were not deciding factors in the success of the implementation. Stakeholders’ trust in the data, the model and its outputs (evidence strength and quality) was important but insufficient to ensure the modelling results were successfully implemented. This is reflected in the literature which shows that populating models with reliable and valid data is critical [53]; however, there is no guarantee for the uptake of modelling outputs even for the best models [15]. Contrary to stakeholders’ beliefs, a lack of available resources did not impede service changes based on the modelling in our study. A lack of dedicated resources is not a commonly reported barrier for implementing modelling results in the literature. The role of stakeholders’ autonomy and control over decision making in implementing modelling results remains unclear. The authors recommend that future revisions of the CFIR framework should consider inclusion of the degree to which stakeholders have autonomy and control to influence decisions.

This qualitative evaluation has several limitations. Firstly, this study was a pragmatic qualitative study based on a sub-set of CFIR constructs. Future studies should consider a mixed methods approach examining all CFIR constructs to gain a more comprehensive understanding of a wider range of factors that influence the uptake of healthcare modelling results. Secondly, the current study did not examine the role of the cost of undertaking the modelling as the modelling costs were funded by project grant funds. Thirdly, the participating stakeholders' views may not have been representative of the views of their whole services or health districts. Finally, we conducted the focus groups at the health district level, rather than at the service level, to reduce staff burden. At sites with more than one participating service, we found it challenging at times to determine whether the stakeholders’ comments referred to one or both of its participating services. It should be noted that the 10-month follow-up period in this study to conduct the second round of focus groups was planned to align with the annual budget cycles and the business case outcomes. This short timeframe may be insufficient to capture the simulation model results being put into practice as there is often a lag time between modelling and the implementation of its results [18].

The costs of the simulation modelling implementation strategy were found to be consistent across the participating services (Additional file 5). It was not surprising that the initial stage of the implementation strategy incurred the large majority of costs as it involved developing the model, including data collection and validation. The use of prospectively collected data to cost the implementation activities, via detailed self-report activity logs, was a strength of this study. These costs are commonly ignored in evaluations of implementation strategies in health care [54, 55]. They nonetheless represent real costs which are important for decision makers looking to develop a simulation model locally and use its results to inform planning of a new service or changes to an existing service [56].

There were limitations of the costing analysis. Firstly, as this analysis relates to modelling activities within a relatively homogenous group of outpatient services, with consistencies in data availability and service structures, the costs associated with the simulation modelling implementation may not be able to be generalised to other outpatient services. Further, as the modelling involved cloning and adapting a previously validated model, it is likely that developing and refining a new simulation model would incur additional costs.

## Conclusions

While there is a wealth of data available within the hospital and health systems to help inform decisions, it is often not in a format that is ready to use for decision making. Advances in computing have meant that data-driven techniques are able to transform existing hospital data into evidence to help inform service planning and decisions on resource allocation. Simulation modelling, tailored to local healthcare contexts, may be a step towards enabling decision makers to plan the most efficient scale and configuration of services to manage service demand and to keep waitlists under control over the medium term. Simulation modelling is a complex undertaking, and stakeholders may have little or no experience with simulation modelling. Using an implementation science approach to examine how and why key decision makers adopt modelling, our findings can be used to inform implementation strategies and may make this complex tool more accessible to decision makers for health service planning.

## Additional files

Additional file 1: Proportion of patients waiting longer than clinically recommended on specialist orthopaedic and neurosurgical outpatient waiting lists. (DOCX 101 kb)

Additional file 2: Standards for Reporting Implementation Studies: the StaRI checklist for completion. (DOCX 212 kb)

Additional file 3: Staff time collection template. (DOCX 58 kb)

Additional file 4: Economic evaluation of enacting the modelled changes to service delivery within one outpatient service: case study. (ZIP 152 kb)

Additional file 5: Time and costs of implementation activities, by position type, implementation strategy stage and project site. (DOCX 28 kb)

## Data Availability

The datasets generated and analysed during the current study are not publicly available due to them containing information that could compromise research participant privacy/consent.

## Abbreviation

CFIR: Consolidated framework for implementation research

## References

[1] Vos T, Flaxman AD, Naghavi M, Lozano R, Michaud C, Ezzati M (2012). Years lived with disability (YLDs) for 1160 sequelae of 289 diseases and injuries 1990-2010: a systematic analysis for the Global Burden of Disease Study 2010. Lancet..

[2] Arthritis and Osteoporosis Victoria (2013). A problem worth solving.

[3] Rymaszewski LA, Sharma S, McGill PE, Murdoch A, Freeman S, Loh T (2005). A team approach to musculo-skeletal disorders. Ann R Coll Surg Engl.

[4] Comans T, Raymer M, O'Leary S, Smith D, Scuffham P (2014). Cost-effectiveness of a physiotherapist-led service for orthopaedic outpatients. J Health Serv Res Policy.

[5] Standfield L, Comans T, Raymer M, O'Leary S, Moretto N, Scuffham P (2016). The efficiency of increasing the capacity of physiotherapy screening clinics or traditional medical services to address unmet demand in orthopaedic outpatients: a practical application of discrete event simulation with dynamic queuing. Appl Health Econ Health Policy.

[6] Pitt M, Monks T, Crowe S, Vasilakis C (2016). Systems modelling and simulation in health service design, delivery and decision making. BMJ Qual Saf.

[7] Drummond M, Jonsson B, Rutten F (1997). The role of economic evaluation in the pricing and reimbursement of medicines. Health Policy.

[8] Cohen DJ, Reynolds MR (2008). Interpreting the results of cost-effectiveness studies. J Am Coll Cardiol.

[9] Budget Impact Analysis [online] York (2016). York Health Economics Consortium.

[10] Mielczarek B (2016). Review of modelling approaches for healthcare simulation. Oper Res Decis.

[11] Salleh S, Thokala P, Brennan A, Hughes R, Booth A (2017). Simulation modelling in healthcare: an umbrella review of systematic literature reviews. PharmacoEconomics..

[12] Bayer S, Brailsford SC, Bolt T (2009). Examining the role of simulation models in health planning.

[13] Hamrock E, Paige K, Parks J, Scheulen J, Levin S (2013). Discrete event simulation for healthcare organizations: a tool for decision making. J Healthc Manag.

[14] Marshall DA, Burgos-Liz L, IJzerman MJ, Osgood ND, Padula WV, Higashi MK (2015). Applying dynamic simulation modeling methods in health care delivery research-the SIMULATE checklist: report of the ISPOR simulation modeling emerging good practices task force. Value Health.

[15] Günal MM, Pidd M (2010). Discrete event simulation for performance modelling in health care: a review of the literature. J Simul.

[16] Comans TA, Chang AT, Standfield L, Knowles D, O’Leary S, Raymer M (2017). The development and practical application of a simulation model to inform musculoskeletal service delivery in an Australian public health service. Oper ResHealth Care.

[17] Wilson JC (1981). Implementation of computer simulation projects in health care. J Oper Res Soc.

[18] Fone D, Hollinghurst S, Temple M, Round A, Lester N, Weightman A (2003). Systematic review of the use and value of computer simulation modelling in population health and health care delivery. J Public Health Med.

[19] Brailsford SC, Harper PR, Patel B, Pitt M (2009). An analysis of the academic literature on simulation and modelling in health care. J Simul.

[20] Katsaliaki K, Mustafee N, Mustafee N (2016). Applications of simulation within the healthcare context. Operational research for emergency planning in healthcare: volume 2.

[21] Katsaliaki K, Mustafee N (2011). Applications of simulation within the healthcare context. J Oper Res Soc.

[22] Lane DC, Monefeldt C, Husemann E (2003). Client involvement in simulation model building: hints and insights from a case study in a London hospital. Health Care Manag Sci.

[23] Brailsford SC, Bolt TB, Bucci G, Chaussalet TM, Connell NA, Harper PR (2013). Overcoming the barriers: a qualitative study of simulation adoption in the NHS. J Oper Res Soc.

[24] Monks T, Pearson M, Pitt M, Stein K, James MA (2015). Evaluating the impact of a simulation study in emergency stroke care. Oper Res Health Care.

[25] Brailsford S (2005). Overcoming the barriers to implementation of operations research simulation models in healthcare. Clin Invest Med.

[26] Jun JB, Jacobson SH, Swisher JR (1999). Application of discrete-event simulation in health care clinics: a survey. J Oper Res Soc.

[27] Harper PR, Pitt MA (2004). On the challenges of healthcare modelling and a proposed project life cycle for successful implementation. J Oper Res Soc.

[28] Queensland Health (2016). Queensland reporting hospitals: quarterly information for specialist outpatient at 1 October 2016.

[29] Queensland Health (2017). Queensland reporting hospitals: quarterly information for specialist outpatient at 1 January 2017.

[30] Moretto N, Comans T, Chang A, O’Leary S, Knowles D, Standfield L (2018). Simulation modelling to support service planning in specialist Neurosurgical and Orthopaedic outpatient services at Gold Coast Health.

[31] Moretto N, Comans T, Chang A, O’Leary S, Knowles D, Standfield L (2018). Simulation modelling to support service planning in specialist orthopaedic and neurosurgical outpatient services at Cairns and Hinterland Hospital and Health Service.

[32] Moretto N, Comans T, Chang A, O’Leary S, Knowles D, Standfield L (2018). Simulation modelling to support service planning in specialist orthopaedic outpatient services at West Moreton Hospital and Health Service.

[33] Damschroder LJ, Aron DC, Keith RE, Kirsh SR, Alexander JA, Lowery JC (2009). Fostering implementation of health services research findings into practice: a consolidated framework for advancing implementation science. Implement Sci.

[34] QSR International Pty Ltd (2012). NVivo qualitative data analysis software.

[35] Sopcak N, Aguilar C, O'Brien MA, Nykiforuk C, Aubrey-Bassler K, Cullen R (2016). Implementation of the BETTER 2 program: a qualitative study exploring barriers and facilitators of a novel way to improve chronic disease prevention and screening in primary care. Implement Sci.

[36] Glaser BG, Strauss AL. Discovery of Grounded Theory. New York: Routledge; 1999.

[37] Proctor E, Silmere H, Raghavan R, Hovmand P, Aarons G, Bunger A (2011). Outcomes for implementation research: conceptual distinctions, measurement challenges, and research agenda. Admin Pol Ment Health.

[38] Damschroder LJ, Lowery JC (2013). Evaluation of a large-scale weight management program using the consolidated framework for implementation research (CFIR). Implement Sci.

[39] Thorwarth M, Arisha A (2009). Application of discrete-event simulation in health care: a review.

[40] Forsberg HH, Aronsson H, Keller C, Lindblad S (2011). Managing health care decisions and improvement through simulation modeling. Qual Manag Health Care.

[41] Steins K, Persson F (2015). Identifying factors for successful implementation of simulation modeling in healthcare. Int JPrivacy Health Inform Manage.

[42] Jahangirian M, Taylor SJE, Eatock J, Stergioulas LK, Taylor PM (2015). Causal study of low stakeholder engagement in healthcare simulation projects. J Oper Res Soc.

[43] Queensland Government Statistician’s Office, Queensland Treasury (2018). Queensland Government population projections.

[44] Eldabi T. Implementation issues of modeling healthcare problems: misconceptions and lessons. In: Rossetti MD, Hill RR, Johansson B, Dunkin A, Ingalls RG (eds) Proceedings of the 2009 Winter Simulation Conference. Piscataway: Institute of Electrical and Electronics Engineers; 2009, pp 1831–1839.

[45] Aharonson-Daniel L, Paul RJ, Hedley AJ (1996). Management of queues in out-patient departments: the use of computer simulation. J Manag Med.

[46] Alkaabi R, Halim AAE, Mahmoud S (2006). Improving resource allocation efficiency in health care delivery systems. 2006 Canadian Conference on Electrical and Computer Engineering; 2006 May.

[47] Cochran JK, Bharti A (2006). Stochastic bed balancing of an obstetrics hospital. Health Care Manag Sci.

[48] Monks T (2016). Operational research as implementation science: definitions, challenges and research priorities. Implement Sci.

[49] Dodgson M, Gann DM, Salter A (2007). The impact of modelling and simulation technology on engineering problem solving. Tech Anal Strat Manag.

[50] Elkhuizen SG, Das SF, Bakker PJM, Hontelez JAM (2007). Using computer simulation to reduce access time for outpatient departments. Qual Saf Health Care.

[51] Heinrichs M, Beekman R, Limburg M (2000). Simulation to estimate the capacity of a stroke unit. Stud Health Technol Inform.

[52] Rytilä JS, Spens KM (2006). Using simulation to increase efficiency in blood supply chains. Manag Res News.

[53] Hvitfeldt-Forsberg Helena, Mazzocato Pamela, Glaser Daniel, Keller Christina, Unbeck Maria (2017). Staffs’ and managers’ perceptions of how and when discrete event simulation modelling can be used as a decision support in quality improvement: a focus group discussion study at two hospital settings in Sweden. BMJ Open.

[54] Hoomans T, Evers SM, Ament AJ, Hubben MW, van der Weijden T, Grimshaw JM (2007). The methodological quality of economic evaluations of guideline implementation into clinical practice: a systematic review of empiric studies. Value Health.

[55] Vale L, Thomas R, MacLennan G, Grimshaw J (2007). Systematic review of economic evaluations and cost analyses of guideline implementation strategies. Eur J Health Econ.

[56] Hoomans T, Severens JL (2014). Economic evaluation of implementation strategies in health care. Implement Sci.

