# Supplementary material for: Implementation of simulation modelling to improve service planning in specialist orthopaedic and neurosurgical outpatient services
Source: Implement Sci. 2019 Aug 9;14:78. doi: 10.1186/s13012-019-0923-1 (PMC6688348; doi:10.1186/s13012-019-0923-1)
Supplement: Supplementary file 1 — Proportion of patients waiting longer than clinically recommended on specialist orthopaedic and neurosurgical outpatient waiting lists. (DOCX 101 kb) [file 13012_2019_923_MOESM1_ESM.docx]

Additional file 1

**Proportion of patients waiting longer than clinically recommended on specialist orthopaedics and neurosurgical outpatient waiting lists**

Table S1. Baseline proportion of patients waiting longer than clinically recommended on specialty (orthopaedics and neurosurgical) outpatient waiting lists by outpatient clinical urgency category as at 1 October 2016 (Sites A and B) and 1 January 2017 (Site C)

| **Specialty** | **Outpatient urgency category*** | | | | **Date** | **Source** |
| --- | --- | --- | --- | --- | --- | --- |
|  | **Cat 1** | **Cat 2** | **Cat 3** | **Total** |  |  |
| Orthopaedics |  |  |  |  |  |  |
| Site A | 43% | 85% | 65% | 74% | 01/10/2016 | Queensland health (2016)^1^ |
| Site B | 6% | 4% | 3% | 3% | 01/10/2016 | Queensland health (2016)^1^ |
| Site C | 8% | 42% | 12% | 17% | 01/01/2017 | Queensland health (2017)^2^ |
| Mean (all sites) | 19% | 44% | 27% | 31% |  |  |
| Neurosurgical |  |  |  |  |  |  |
| Site A | 43% | 94% | 66% | 78% | 01/10/2016 | Queensland health (2016)^1^ |
| Site B | NS | NS | NS | NS |  |  |
| Site C | 26% | 70% | 2% | 19% | 01/01/2017 | Queensland health (2017)^2^ |
| Mean (all sites) | 35% | 82% | 34% | 49% |  |  |
| Overall mean | 25% | 59% | 30% | 38% |  |  |

Cat: category; NS: no service

* Outpatients are categorised as urgent (category 1), semi-urgent (category 2) and non-urgent (category 3) with recommended timeframes for an initial outpatient consultation within 30, 90, and 365 days, respectively.

**References**

1. Queensland Health. Queensland reporting hospitals: quarterly information for specialist outpatient at 1 October 2016. 2016 [Available from: <http://www.performance.health.qld.gov.au/hospitalperformance/op-main.aspx?hospital=99999> [verified 1 December 2016].
2. Queensland Health. Queensland reporting hospitals: quarterly information for specialist outpatient at 1 January 2017. 2017 [Available from: <http://www.performance.health.qld.gov.au/hospitalperformance/op-main.aspx?hospital=99999> [verified 1 March 2017].
