# Supplementary material for: Implementation of simulation modelling to improve service planning in specialist orthopaedic and neurosurgical outpatient services
Source: Implement Sci. 2019 Aug 9;14:78. doi: 10.1186/s13012-019-0923-1 (PMC6688348; doi:10.1186/s13012-019-0923-1)
Supplement: Supplementary file 3 — Staff time collection template. (DOCX 58 kb) [file 13012_2019_923_MOESM3_ESM.docx]

Additional file 3

**Staff time collection template**

The below template was administered to project staff and relevant stakeholders to record the time spent (in hours) on key activities related to the three stages of the simulation modelling implementation strategy.

| Current position | Salary | | Time spent on project activities ^1^ | | | |
| --- | --- | --- | --- | --- | --- | --- |
|  | Pay level | Pay stream | Stage 1 | Stage 2 | Stage 3 |  |
|  |  | Executive / Medical / Nursing / Allied health / Admin | Hours  (min. – max.) | Hours  (min. – max.) | Hours  (min. – max.) |  |
| *Example, Director of physiotherapy* | *HP6* | *Allied Health* | *5 hours (2 – 7)* | *5 hours (2 – 7)* | *5 hours (2 – 7)* |  |
|  |  |  |  |  |  |  |
|  |  |  |  |  |  |  |
|  |  |  |  |  |  |  |
|  |  |  |  |  |  |  |

^1^ Stage 1 of the project included stakeholder engagement, model development and initial modelling results. Stage 2 included exploration of feasible scenarios. Stage 3 involved making changes to service delivery. Refer to the manuscript for more details.
