# Supplementary material for: Implementation of simulation modelling to improve service planning in specialist orthopaedic and neurosurgical outpatient services
Source: Implement Sci. 2019 Aug 9;14:78. doi: 10.1186/s13012-019-0923-1 (PMC6688348; doi:10.1186/s13012-019-0923-1)
Supplement: Supplementary file 4 — Economic evaluation of enacting the modelled changes to service delivery within one outpatient service: case study. (ZIP 152 kb) [file 13012_2019_923_MOESM4_ESM.zip › Economic evaluation of enacting the modelled changes to service delivery within one outpatient service.docx]

# Additional file 4

# Economic evaluation of enacting the modelled changes to service delivery within one outpatient service: Case study

## Context

The value of simulation modelling in healthcare is unclear, which is largely due to limited economic evaluations of modelling implementation [1-5]. To help address this gap in the literature, we aimed to perform an economic evaluation of a scenario involving the adoption of recommendations from a simulation model for the re-design of a musculoskeletal outpatient service.

## Methods

We conducted an economic evaluation of a scenario in which a simulation model was developed to inform service planning and the model’s recommendations for musculoskeletal service re-design were adopted in one of the participating outpatient services. We selected one of the participating services at Site C as the basis for the case study as the modelling recommendations were included in a business case that was approved by the health district’s executive for the 2017/18 financial year. The analysis was conducted from the Australian health system perspective.

### Costs associated with the implementation strategy (including developing the model)

We collected costs for all three stages of the simulation modelling implementation strategy: (i) Stakeholder engagement, model development and presentation of initial modelling results (Stage 1); (ii) exploration of feasible scenarios (Stage 2); and (iii) changes to service delivery (Stage 3). Project team members and relevant stakeholders completed a self-report activity log at the end of each stage estimating the number of hours spent on activities during each stage from 1 June 2016 to 30 September 2017 (see Additional file 3). Staff time was valued using 2016–17 financial year salary data including employer on-costs. Where salary levels were unknown, the upper band of the pay scale was used. Contractor time and costs were taken directly from the invoices received for activities related to the project. We excluded costs associated with the qualitative evaluation, overheads, computers and modelling software licences.

### Costs associated with service re-design

We included the cost of changes to the delivery of musculoskeletal outpatient services at the example site. These costs were for an additional investment for increased staffing in the physiotherapist-led service for the 12-month period (2017/18 financial year). The increased staffing was aimed at addressing increased scale of services delivered and the proportion of referrals directed to physiotherapist-led services recommended in the feasible scenario of the model. These costs were extracted from the submitted business case (2017/18 financial year) and reflect actual funds committed by the health district’s executive.

### Outcomes

The primary outcome measure was the number of long waits avoided at the end of the 12-month period. This was defined as the number of patients who had been waiting longer than clinically recommended for an initial consultation based on their clinical urgency category (i.e. urgent, semi-urgent and non-urgent referrals were recommended to be seen within 30, 90, and 365 days, respectively). We calculated the number of long waits avoided as the difference in the number of patients projected to breach target wait times if the service continued unchanged (modelled base case), compared to if service changes were enacted based on modelling recommendations (modelled feasible scenario). This data was calculated from the outputs of the simulation model (i.e. projected waiting lists multiplied by the complement of the proportion of patients seen within clinically recommended timeframes).

### Decision makers’ willingness to pay

The value decision makers place on avoided long waits can be estimated from publicly announced funding decisions specifically to address specialist outpatient waiting lists. We examined recent Queensland Health policy announcements to explore the state government’s willingness to pay for outpatient initiatives to target specialist outpatient long waits. Estimates of the decision makers’ willingness to pay was calculated as the expenditure divided by the number of longs waits.

## Results

Costs and outcomes of the implementation of the simulation model at one service are presented in Table S1. The costs associated with the development of the simulation model and implementation strategy at this service was estimated to be between AU$34,287 to AU$35,636. A breakdown of these costs is provided in Additional file 5. This represented a very small proportion (<5%) of the overall total costs of implementing the model and enacting the recommended service changes.

The simulation model projections of the waiting list and the number of long wait patients at the end of a 12-month period for the base case and the feasible scenario are provided in Appendix A. The model predicts that in order to reduce the number of patients breaching their clinically recommended wait time targets from 2,415 patients to 72 patients (i.e. 2,343 long wait cases avoided) at the end of a 12-month period, the combined outpatients service would need to almost double the number of new patients seen over this time. To achieve this, an additional 1,344 new patients would need to be seen in this period; with the feasible scenario suggesting that the vast majority of these patients could be seen in the physiotherapist-led service.

A business case was prepared to increase staffing in the physiotherapist-led service to achieve this increased new patient activity over the 12-month period. The business case was approved by the health district’s executive and funding was committed for 6.4 full-time equivalent (FTE) positions above current staffing positions for a 12-month period. This represented an additional investment of AU$838,000, which is additional to the service’s current operating budget. This investment was proposed to provide additional advanced physiotherapist-led outpatient assessments (2.4 FTE advanced physiotherapists), downstream multidisciplinary care (3 FTE multidisciplinary team) and administrative support (1 FTE).

The combined costs associated with the development of the simulation model and implementation strategy, and enacting the recommended service changes for a 12-month period, was therefore estimated at a total cost of AU$873,636.

Table S1. Cost and outcomes associated with developing the simulation model and enacting service changes based on the model’s results

|  | **Business as usual** | **Implement modelling and enact service changes** | **Difference** | **Ref** | **Source/Calculation** |
| --- | --- | --- | --- | --- | --- |
| **Costs** |  |  |  |  |  |
| *Development of the simulation model and implementation strategy* |  |  |  |  |  |
| Stage 1: Engage stakeholders, develop model, and present initial modelling results | - | $27,811 | $27,811 | A | Project data collection |
| Stage 2: Explore feasible scenarios | - | $4,564 | $4,564 | B | Project data collection |
| Stage 3: Prepare to make service delivery changes | - | $3,261 | $3,261 | C | Project data collection |
| Sub-total | - | $35,636^1^ | $35,636 | D | Project data collection |
|  |  |  |  |  |  |
| *Service re-design (following the simulation model’s results and recommendation)* |  |  |  |  |  |
| Operating costs for musculoskeletal outpatient services (12 months) | BAU | BAU + $838,000 | $838,000 | E | Business case |
| Staffing (Physiotherapist-led service), FTE | 9.1 | 15.5 | 6.4^3^ | F | Business case |
|  |  |  |  |  |  |
| *Overall total costs* | *BAU* | *BAU + $873,636* | *$873,636* | G | Calculation (D + E) |
|  |  |  |  |  |  |
| **Outcomes** |  |  |  |  |  |
| *New patients seen (Model)^2^* |  |  |  |  |  |
| New patients to be seen over 12 months (Neurosurgeon-led) | 1,020 | 1,068 | 48 | H | Model |
| New patients to be seen over 12 months (Physio-led)^4^ | 540 | 1,836 | 1,296 | I | Model |
| *Total new patients* | *1,560* | *2,904* | *1,344* | J | Model |
|  |  |  |  |  |  |
| *Waiting list* |  |  |  |  |  |
| *Projected number of patients on the waiting list at 12 months (June 2018)* | *2,713* | *1,241* | *1,472* | K | Model (Appendix A) |
|  |  |  |  |  |  |
| *Number of long wait patients* |  |  |  |  |  |
| *Projected number of long waits at 12 months (June 2018) ^5^* | *2,415* | *72* | *2,343* | L | Model (Appendix A) |
|  |  |  |  |  |  |
| **Cost per outcome** |  |  |  |  |  |
| Cost per long wait case avoided in physiotherapist-led service |  |  | $373 | M | Calculation (G ÷ L) |
| Cost per additional patient seen in physiotherapist-led service |  |  | $674 | N | Calculation (G ÷ I) |

BAU: Business as usual operating budget; FTE: full-time equivalent. Costs are in Australian dollars.

^1^ This figure provide a conservative estimate as it represents the upper cost estimate. The lower estimate was AU$ 34,287.

^2^ The model conservatively assumed a 2.1% growth rate in the number of patients presenting per year.

^3^ The business case requested 7.4 FTE, but 0.5 FTE multidisciplinary team and 0.5 FTE administration officer was not funded. Funds were committed for 6.4 FTE (i.e. 2.4 FTE physiotherapist clinical leads to provide additional advanced physiotherapist-led outpatient assessments; 3 FTE multidisciplinary team to provide downstream multidisciplinary care; and 1 FTE in administrative support).

^4^ The business case noted 900 new patients (BAU) and activity target of 2,400 new patients with the service changes, resulting in a difference of 1,500 new patients to be seen over 12 months in the physiotherapist-led service.

^5^ This snapshot figure from the simulation model may represent a conservative estimate of the number of long wait cases avoided as additional long wait patients may have been seen during this time.

This total cost divided by the projected 2,343 long wait cases avoided at 12 months, represents an estimated cost of $373 per long wait case avoided. The executive’s approval to commit additional funds to change the scale and professional mix of outpatient services, compared to business as usual, indicates that the costs were acceptable at the willingness to pay of the decision makers at a health district level.

An examination of the recent Queensland Health policy announcements revealed that the state government was willing to spend an additional AU$361 million over four years on a range of initiatives to target 104,000 specialist outpatient long waits (Table S2). Queensland Health’s investment in specialist outpatient services was directly related to the unacceptable number of long waits. The state government’s willingness to pay can therefore be inferred to be $3,471 per long wait case avoided. It is important to note that this figure represents investment in outpatient services only, and does not reflect the costs associated with ongoing treatment that may occur outside of the outpatient setting.

Table S2. Queensland Health funding decision taken to reduce long waits (2016)

|  | **Unit** | **Ref** | **Source** |
| --- | --- | --- | --- |
| *Queensland Health specialist outpatient strategy (2016)* [6] | | | |
| Amount invested over 4 years (AU$) | $361M | A | Queensland Health (2016) [6] |
| Number of long wait cases^1^ | 104,000 | B | Queensland Health (2016) [6] |
| *Willingness to pay (inferred)* |  |  |  |
| Cost per long wait case (AU$) | $3,471 | C | A ÷ B |

^1^ Number of long wait cases at the time of the announcement

It can be inferred that undertaking simulation modelling and enacting the recommended service changes to optimise service delivery has the potential to reduce long waits at approximately one ninth of the health system funder’s willingness to pay.

## Discussion

This case study describes the costs and outcomes of the implementation of the simulation model. The overall cost to implement and enact changes from the simulation model was $873,636 for a 12-month period. This case study showed that this investment had a positive benefit for patients, compared to business as usual, with 97% of patients that were projected to breach wait time targets were predicted to be seen within the clinically recommended timeframes as a result of the increased activity. The cost of $373 per long wait case avoided, was shown to be acceptable at the willingness to pay of the decision makers at the district level.

Stakeholders were keen to know whether the costs associated with the implementation of the simulation model to inform service planning represented value for money. Addressing the problem of patients waiting longer than clinically recommended for their initial outpatient appointment is a priority for the state government [6, 7]. This is reflected in the state government’s willingness to pay for a long wait avoided, inferred from a recent policy announcement to invest in initiatives to target specialist outpatient long waits [6, 7]. We demonstrated that undertaking simulation modelling and enacting the recommended service changes to optimise service delivery could reduce long waits at one ninth of the health system funder’s willingness to pay. When benchmarked against previous government funding decisions, this represents a high value use of healthcare resources to reduce long waits in this outpatient setting.

Long outpatient waiting times is a systemic issue that extends beyond Queensland, affecting most Australian states and territories [8]. Neurosurgical and orthopaedic outpatient services across Australia that would likely benefit from the simulation model implementation are those with long wait times, indicative of sub-optimal scale or professional mix of services, and those in need of service re-design. The value of a simulation model implementation may vary between musculoskeletal outpatient services based on individual service’s current and projected future demand-capacity characteristics and the type of changes being enacted (e.g. increasing physiotherapist-led activity; increasing medical specialist-led activity; implementing corrective strategies). The health system funder’s willingness to pay is subjective and may vary depending on the jurisdiction as well as the financial, political and socio-economic context in which the outpatient service operates.

There were several limitations of this economic analysis. Firstly, as this analysis is a case study of one outpatient service, the costs and outcomes of the simulation modelling implementation may not be able to be generalised to other outpatient services. Secondly, due to the short timeframe of this study, it was not considered feasible to obtain actual estimates of outcome data for the 12-month period. As such, the outcomes in this analysis, which were sourced from the model’s projections, may be less reliable than actual outcome data. Thirdly, the costs we have presented outline an investment in outpatient services only, and do not reflect the costs associated with any treatment that may occur outside of the outpatient setting, for example, imaging services and pharmaceutical prescriptions.

## Conclusion

Our findings from this case study suggest that taking a pro-active approach to waitlist management would improve patient access to care and at relatively low cost, representing a high value use of health care resources.

## References

1. Brailsford SC, Harper PR, Patel B, Pitt M. An analysis of the academic literature on simulation and modelling in health care. Journal of Simulation. 2009;3(3):130-40.

2. Brailsford SC, Bolt TB, Bucci G, Chaussalet TM, Connell NA, Harper PR, et al. Overcoming the barriers: a qualitative study of simulation adoption in the NHS. The Journal of the Operational Research Society. 2013;64(2):157-68.

3. Monks T, Pearson M, Pitt M, Stein K, James MA. Evaluating the impact of a simulation study in emergency stroke care. Operations Research for Health Care. 2015;6:40-9.

4. Jun JB, Jacobson SH, Swisher JR. Application of discrete-event simulation in health care clinics: A survey. Journal of the Operational Research Society. 1999;50(2):109-23.

5. Pitt M, Monks T, Crowe S, Vasilakis C. Systems modelling and simulation in health service design, delivery and decision making. BMJ Quality & Safety. 2016;25(1):38-45.

6. Queensland Health. Specialist Outpatient Strategy. Improving the patient journey by 2020. Brisbane, Queensland; 2016.

7. $361 million strategy to slash outpatient wait lists [press release]. 6 September 2016.

8. Duckett S. Getting an initial specialists’ appointment is the hidden waitlist: The Conversation; 2018 [Available from: https://theconversation.com/getting-an-initial-specialists-appointment-is-the-hidden-waitlist-99507.
