# Supplementary material for: Implementation of simulation modelling to improve service planning in specialist orthopaedic and neurosurgical outpatient services
Source: Implement Sci. 2019 Aug 9;14:78. doi: 10.1186/s13012-019-0923-1 (PMC6688348; doi:10.1186/s13012-019-0923-1)
Supplement: Supplementary file 4 — Economic evaluation of enacting the modelled changes to service delivery within one outpatient service: case study. (ZIP 152 kb) [file 13012_2019_923_MOESM4_ESM.zip › Simulation model projections of the waiting list, percentage of patients seen within clinically recommended timeframes, and number of long wait cases avoided at the example service.docx]

Appendix A

**Simulation model projections of the waiting list, percentage of patients seen within clinically recommended timeframes, and number of long wait cases avoided at the example service**

Table S1. Simulation model projections of the waiting list, percentage of patients seen within clinically recommended timeframes, and number of long wait cases avoided at the example service (end June 2018)

|  | **Model projections at end June 2018** | | | | | | | | | | |  | | |  |
| --- | --- | --- | --- | --- | --- | --- | --- | --- | --- | --- | --- | --- | --- | --- | --- |
|  | **Base case** | | | | **Feasible scenario** | | | | |  | | |  | | |
|  | **Cat 1** | **Cat 2** | **Cat 3** | **All categories** | | **Cat 1** | **Cat 2** | **Cat 3** | **All categories** | | **Ref** | | | **Source** | |
| Projected number of patients on the waiting list | 0 | 615 | 2,098 | 2,713 | | 1 | 345 | 895 | 1,241 | | A | | | Model | |
| % patient seen within clinically recommended times | 100% | 11% | 11% | NC | | 100% | 80% | 99.7% | NC | | B | | | Model | |
| Projected number of long waits^1^ | 0 | 546 | 1870 | 2,415 | | 0 | 69 | 3 | 72 | | C | | | A x (100% - B) | |

Model assumed a 2.1% growth rate in the number of patients presenting per year. NC: not calculated

^1^ These snapshot figures at June 2018 may represent a conservative estimate of the number of long wait cases avoided as additional long wait patients may have been seen during this time.
