# Supplementary material for: Implementation of simulation modelling to improve service planning in specialist orthopaedic and neurosurgical outpatient services
Source: Implement Sci. 2019 Aug 9;14:78. doi: 10.1186/s13012-019-0923-1 (PMC6688348; doi:10.1186/s13012-019-0923-1)
Supplement: Supplementary file 5 — Time and costs of implementation activities, by position type, implementation strategy stage and project site. (DOCX 28 kb) [file 13012_2019_923_MOESM5_ESM.docx]

Additional file 5

**Time and costs of implementation activities, by position type, implementation strategy stage and project site**

Staff time (hours) spent on implementation activities and associated costs are presented in Tables S1 and S2.

Table S1. Staff time (hours) spent on implementation activities, by position type, implementation strategy stage and project site

| **Specialty** | **Site** | **Stage**^1^ | **Time spent by position type (hours)** | | | | | | | | |
| --- | --- | --- | --- | --- | --- | --- | --- | --- | --- | --- | --- |
|  |  |  | **Academic** | **Admin** | **Contractor** | **Executive** | **Allied health^2^** | **Medical** | **Nursing** | **Travel** | **Total** |
| Orthopaedics | Site A | Stage 1 | 37.2 | 19.2 | 36.7 | 0.0 | 102.8 | 1.3 | 3.0 | 3.6 | **203.7** |
|  |  | Stage 2 | 0.0 | 0.3 | 12.2 | 0.0 | 46.4 | 2.9 | 0.3 | 1.6 | **63.6** |
|  |  | Stage 3 | 0.2 | 0.5 | 0.0 | 0.0 | 58.4 | 3.8 | 1.5 | 0.0 | **64.3** |
|  |  | **Total** | **37.3** | **20.0** | **48.9** | **0.0** | **207.5** | **8.0** | **4.8** | **5.2** | **331.6** |
| Orthopaedics | Site B | Stage 1 | 35.7 | 37.9 | 36.7 | 10.0 | 127.8 | 2.5 | 2.0 | 4.1 | **256.7** |
|  |  | Stage 2 | 0.0 | 1.8 | 12.2 | 5.3 | 42.3 | 1.8 | 1.8 | 2.1 | **67.1** |
|  |  | Stage 3 | 0.2 | 0.0 | 0.0 | 0.0 | 12.5 | 0.0 | 0.0 | 0.0 | **12.7** |
|  |  | **Total** | **35.9** | **39.6** | **48.9** | **15.3** | **182.7** | **4.3** | **3.8** | **6.2** | **336.5** |
| Orthopaedics | Site C | Stage 1 | 34.8 | 77.4 | 37.2 | 2.8 | 93.8 | 1.0 | 0.5 | 3.1 | **250.6** |
|  |  | Stage 2 | 0.5 | 3.8 | 12.2 | 0.8 | 41.0 | 0.0 | 0.0 | 0.1 | **58.3** |
|  |  | Stage 3 | 0.2 | 1.0 | 0.0 | 1.5 | 25.6 | 0.0 | 0.0 | 0.0 | **28.2** |
|  |  | **Total** | **35.5** | **82.1** | **49.4** | **5.0** | **160.4** | **1.0** | **0.5** | **3.2** | **337.1** |
| Neurosurgery | Site A | Stage 1 | 37.2 | 19.2 | 36.7 | 0.0 | 102.1 | 1.3 | 3.0 | 3.6 | **203.1** |
|  |  | Stage 2 | 0.0 | 0.3 | 12.2 | 0.0 | 48.9 | 1.4 | 0.3 | 1.6 | **64.6** |
|  |  | Stage 3 | 0.2 | 0.5 | 0.0 | 0.0 | 52.8 | 0.5 | 0.0 | 0.0 | **53.9** |
|  |  | **Total** | **37.3** | **20.0** | **48.9** | **0.0** | **203.8** | **3.2** | **3.3** | **5.2** | **321.6** |
| Neurosurgery | Site C | Stage 1 | 34.8 | 77.4 | 37.2 | 2.8 | 93.8 | 2.0 | 0.5 | 3.1 | **251.6** |
|  |  | Stage 2 | 0.5 | 3.8 | 12.2 | 0.8 | 43.0 | 0.0 | 0.0 | 0.1 | **60.3** |
|  |  | Stage 3 | 0.2 | 1.0 | 0.0 | 3.0 | 37.3 | 0.0 | 0.0 | 0.0 | **41.4** |
|  |  | **Total** | **35.5** | **82.1** | **49.4** | **6.5** | **174.1** | **2.0** | **0.5** | **3.2** | **353.3** |

Admin: administration

^1^ Stage 1 of the project included stakeholder engagement, model development and initial modelling results. Stage 2 included exploration of feasible scenarios. Stage 3 involved making changes to service delivery*.* ^2^ Includes clinical leads (physiotherapist), directors of physiotherapy, executive directors of allied health, and project team members.

Table S2. Staffing costs of implementation activities, by position type, project staff and project site

| **Specialty** | **Site** | **Stage^1^** | **Cost (AU$) by position type** | | | | | | | | |
| --- | --- | --- | --- | --- | --- | --- | --- | --- | --- | --- | --- |
|  |  |  | **Academic** | **Admin** | **Contractor** | **Executive** | **Allied health^2^** | **Medical** | **Nursing** | **Travel** | **Total** |
| Orthopaedics | Site A | Stage 1 | 2,778 | 1,532 | 2,954 | - | 16,071 | 138 | 184 | 1,715 | **25,372** |
|  |  | Stage 2 | - | 13 | 974 | - | 3,363 | 321 | 19 | 551 | **5,240** |
|  |  | Stage 3 | 12 | 34 | - | - | 4,509 | 422 | 111 | - | **5,088** |
|  |  | **Total** | **2,790** | **1,579** | **3,928** | **-** | **23,942** | **881** | **314** | **2265** | **35,700** |
| Orthopaedics | Site B | Stage 1 | 2,669 | 2,442 | 2,954 | 995 | 17,982 | 275 | 144 | 182 | **27,644** |
|  |  | Stage 2 | - | 97 | 974 | 616 | 3,069 | 193 | 131 | 88 | **5,168** |
|  |  | Stage 3 | 12 | - | - | - | 971 | - | - | - | **983** |
|  |  | **Total** | **2,681** | **2,539** | **3,928** | **1611** | **22,021** | **468** | **275** | **270** | **33,795** |
| Orthopaedics | Site C | Stage 1 | 2,611 | 5,500 | 3,004 | 293 | 15,411 | 110 | 38 | 211 | **27,178** |
|  |  | Stage 2 | 46 | 253 | 974 | 70 | 2,980 | - | - | 11 | **4,334** |
|  |  | Stage 3 | 12 | 64 | 0 | 141 | 2,015 | - | - | - | **2,232** |
|  |  | **Total** | **2,669** | **5,817** | **3,978** | **504** | **20,405** | **110** | **38** | **222** | **33,744** |
| Neurosurgery | Site A | Stage 1 | 2,778 | 1,532 | 2,954 | - | 16,024 | 138 | 184 | 1,715 | **25,325** |
|  |  | Stage 2 | - | 13 | 974 | - | 3,535 | 156 | 19 | 551 | **5,247** |
|  |  | Stage 3 | 12 | 34 | - | - | 3,892 | 55 | - | - | **3,992** |
|  |  | **Total** | **2,790** | **1,579** | **3,928** | **-** | **23,450** | **349** | **202** | **2,265** | **34,564** |
| Neurosurgery | Site C | Stage 1 | 2,611 | 5,500 | 3,004 | 293 | 15,411 | 220 | 38 | 211 | **27,288** |
|  |  | Stage 2 | 46 | 253 | 974 | 70 | 3,131 | - | - | 11 | **4,486** |
|  |  | Stage 3 | 12 | 64 | - | 261 | 2,851 | - | - | - | **3,188** |
|  |  | **Total** | **2,669** | **5,817** | **3,978** | **624** | **21,393** | **220** | **38** | **222** | **34,961** |

All costs are in Australian dollars. Costs were valued using 2016/2017 financial year salary data. Admin: administration.

^1^ Stage 1 of the project included stakeholder engagement, model development and initial modelling results. Stage 2 included exploration of feasible scenarios. Stage 3 involved making changes to service delivery*.* ^2^ Includes clinical leads (physiotherapist), directors of physiotherapy, executive directors of allied health, and project team members.
